# Lieber professionell als spirituell. Die Kommunikation von Angeboten der Achtsamkeitsförderung im Hochschulsetting

**DOI:** 10.1007/s11553-021-00920-2

**Published:** 2021-11-24

**Authors:** Inga Werneburg, Doreen Reifegerste, Birgit Jäpelt

**Affiliations:** 1 Universität Erfurt, Erfurt, Deutschland; 2 Fakultät Gesundheitswissenschaften, Universität Bielefeld, Universitätsstr. 25, 33615 Bielefeld, Deutschland

**Keywords:** Achtsamkeit, Studierendengesundheit, Stressbewältigung, Kommunikationskampagne, Zielgruppenspezifische Kommunikation, Mindfulness, Student health, Stress management, Communication campaign, Targe-group torientated communication

## Abstract

**Hintergrund:**

Angebote zur Achtsamkeitsförderung durch Stressbewältigung sind zunehmend ein wichtiger Teil des Gesundheitsmanagements in Hochschulen geworden. Der Bedarf dafür hat sich gerade in der COVID-19-bedingten („coronavirus disease 2019“) Pandemiesituation deutlich verstärkt. Allerdings werden die Angebote von den Studierenden bislang noch sehr zögerlich in Anspruch genommen, obwohl die positiven Effekte von Achtsamkeitstrainings bereits vielfach belegt sind.

**Ziel der Arbeit:**

Ziel unserer Untersuchung war es daher, Vorstellungen der Achtsamkeitspraxis sowie Zugangsbarrieren zu erfassen, um daraus zielgruppenspezifische Kommunikationsstrategien abzuleiten.

**Methode:**

Es wurden männliche und weibliche Studierende (mit wenig und mit viel Erfahrung in Achtsamkeitstrainings) qualitativ interviewt.

**Ergebnisse:**

Es zeigt sich, dass die Studierenden eine weltanschaulich neutrale und evidenzbasierte Rahmung der Angebote bevorzugen. Eine Betonung des spirituellen Hintergrunds der Achtsamkeitstrainings scheint v. a. den Einsteigern den Zugang zu erschweren (auch wenn sie für die Fortgeschrittenen ein relevanter Bestandteil ist). Zudem ist es den Interviewten wichtig, dass die Kommunikation die Relevanz für die Bewältigung akademischer und beruflicher Herausforderungen aufzeigt, ohne die Angebote als Maßnahme zur Leistungssteigerung zu bewerben.

**Schlussfolgerung:**

Auch wenn die Achtsamkeitspraktiken spirituell verankert sind, stehen Einsteiger diesen Aspekten eher skeptisch gegenüber. Dies deckt sich mit allgemeineren Diskussionen zur Einführung von Achtsamkeitsangeboten an Hochschulen. Darüber hinaus lassen sich Hinweise zur Auswahl von Multiplikator:innen (authentische Vermittler:innen) und Botschaftsstrategien (vielseitige Fallbeispiele) ableiten.

## Abstract (en)

**Background:**

Mindfulness training has become an increasingly important part of health management at universities. The need for this type of training has been reinforced by the massive limitations of presence education and social contacts due to coronavirus disease 2019 (COVID-19) prevention. However, students are still quite reluctant to take part in such training, although their positive effects have been repeatedly proven.

**Objectives:**

Thus, the goal of our research was to examine perceptions and barriers among university students, and to deduce strategies for target-group oriented communication for mindfulness training.

**Methods:**

We conducted qualitative interviews with male and female students with low and high experience in mindfulness training.

**Results:**

It was shown that students prefer an ideologically neutral and evidence-based framing of the trainings. Emphasis of the spiritual background seems to limit the access for inexperienced users (although they are a relevant part for experienced users). In addition, it is important for the interviewed students that the advertisement for the mindfulness training depicts the relevance for academic and professional challenges without selling them as tools for performance enhancement.

**Conclusions:**

Although the roots of mindfulness training is spiritual, inexperienced users are skeptical about these aspects. This corresponds with more general discussions about the introduction of mindfulness training at universities. Furthermore, evidence for the selection of gatekeepers (authentic intermediaries) and the selection of message strategies (diverse exemplars) can be deduced.

## Hintergrund und Fragestellung

### Relevanz

Bei Studierenden werden immer mehr stressbedingte Erkrankungen wie Depressionen, Angststörungen und chronische Erschöpfung diagnostiziert [4, 12]. Diese Entwicklung hat sich aufgrund der massiven Einschränkungen der Präsenzlehre und sozialer Kontakte zur COVID-19-Prävention („coronavirus disease 2019“) deutlich verstärkt [7]. Achtsamkeitskompetenzen könnten dabei für Studierende wichtig sein, um ihre Stressbewältigung und Emotionsregulation zu fördern [13], denn sie tragen nicht nur zur besseren Bewältigung des Studiums bei, sondern auch zur Stärkung der Resilienz im (späteren) Arbeitsleben [16]. Somit ergibt sich eine nachhaltige akademische (Selbst‑)Bildungserfahrung.

Eine Vielzahl an Studien belegt bereits die Wirksamkeit von Achtsamkeitstrainings (für eine Metaanalyse s. [17]), worunter wir Übungen verstehen mit dem Ziel, „ein nicht-wertendes Gewahrsein im gegenwärtigen Moment, Geduld, Vertrauen, Akzeptanz, Loslassen, Sanftmut, Großzügigkeit, Empathie und Dankbarkeit“ zu erlernen [15]. Die Studien kommen zu dem Ergebnis, dass Interventionen mit achtsamkeitsbasierten Verfahren zu besserer Stressbewältigung, Emotionsregulation, Konzentration und Aufmerksamkeit, Impulskontrolle sowie zu einer Steigerung der kognitiven Leistungsfähigkeit führen [1]. Jedoch untersuchen die vorhandenen Studien [17] meist nur die Wirksamkeit der Angebote. Unser Fokus richtet sich jedoch auf Vorstellungen der Studierenden, v. a. auf diejenigen, die die Akzeptanz und Teilnahme verhindern.

Studierende scheinen noch viele Vorbehalte gegenüber diesen Angeboten zu haben, denn ein Großteil fühlt sich zwar gestresst, aber nur ein geringer Anteil (13 %) nimmt Angebote zum Umgang mit Stress, Prüfungsangst und *Achtsamkeit* in Anspruch [8]. Dies lässt sich u. a. damit erklären, dass Studierende diese Angebote gar nicht wahrnehmen oder sie aus einem Mangel an Interesse, Bereitschaft und Zeit nicht nutzen [11]. Es besteht somit eine Diskrepanz zwischen dem Bedarf an und der Inanspruchnahme von Unterstützungsangeboten [16]. Um diese Lücke zu schließen, braucht es wirksame Strategien in der Hochschulkommunikation [11].

Unsere zentrale Fragestellung lautet daher, wie Achtsamkeitsangebote an Hochschulen kommuniziert werden können, damit Studierende sie als Möglichkeiten zur Förderung ihrer Gesundheit wahrnehmen, akzeptieren und in Anspruch nehmen. Spezifisch möchten wir untersuchen, welche Vorstellungen Studierende mit Achtsamkeitsangeboten verbinden und welche Barrieren und Zugangsmöglichkeiten sie wahrnehmen. Diese Erkenntnisse sollen dazu dienen, geeignete Kommunikationsstrategien zur Vermittlung einer achtsamen Lebensführung im Hochschulsetting zu entwickeln.

### Achtsamkeitsförderung im Setting Hochschule

Angebote zur Achtsamkeitsförderung und deren Evaluation finden sich bereits an vielen deutschen Hochschulen. Für einen Überblick s. z. B.: www.achtsamehochschulen.de oder: www.netzwerk-achtsamkeit-in-der-bildung.de. So erforschte z. B. das Thüringer Modellprojekt Achtsame Hochschulen in der digitalen Gesellschaft die Vermittlung von Achtsamkeit als Metakompetenz [2].

Achtsamkeitstrainings werden u. a. im Rahmen von Lehrveranstaltungen, Hochschulsportkursen, als Teil eines Studiengangs oder extracurricular angeboten [14]. Achtsamkeitselemente einschließend, gibt es darüber hinaus Schulungsangebote zur Stressbewältigung, zum Zeitmanagement oder zur Persönlichkeitsentwicklung im Rahmen der Karriereentwicklung. Häufige Elemente dieser Angebote sind Atem- und Entspannungstechniken, der sog. Bodyscan, Sitz- und Gehmeditationen oder Yoga-Übungen [10].

Studierende können somit nicht nur in den Prüfungszeiten oder bei Prüfungsangst von den Achtsamkeitspraktiken profitieren, sondern sich allgemein im Bereich der kognitiven und emotionalen Kontrolle verbessern. Gerade weil Studierende als Arbeitnehmer:innen, Multiplikator:innen und Entscheidungsträger:innen der Zukunft angesehen werden können und sich oftmals noch dazu in einer sensiblen Phase der lebenslangen Persönlichkeitsentwicklung befinden, kommt dem Setting Hochschule somit eine Schlüsselrolle zur Förderung der psychischen bzw. geistigen Gesundheit zu. Selbst wenn die Achtsamkeitsangebote im Hochschulsetting vielleicht keinen langfristigen transformativen Prozess ersetzen können, ermöglichen diese zumindest einen Anstoß dafür [13].

Allerdings gibt es bisher trotz der Vielzahl an Initiativen an Hochschulen und der nachgewiesenen Effektivität, noch keinen konkreten Handlungsplan, der *Achtsamkeit* zu einem festen Bestandteil an allen Hochschulen macht [13]. Folglich fehlt es bislang auch an Kommunikationskonzepten oder Strategien, die darauf abzielen, die Wahrnehmung, Akzeptanz und Teilnahme der Studierenden zu erhöhen. Während andere Gesundheitsförderungsbereiche wie Ernährung und Bewegung eine lange Tradition der Kommunikationskampagnen (und dazugehörige Forschung) vorzuweisen haben, liegen bislang für die Kommunikation von Angeboten zur Achtsamkeitsförderung kaum Studienergebnisse vor.

### Kommunikation von Angeboten zur Achtsamkeitsförderung

Die Motive, aus denen heraus sich Menschen Achtsamkeitsangeboten zuwenden, reichen von der Sehnsucht nach Gelassenheit, Freundlichkeit, Zufriedenheit, Offenheit und Akzeptanz, bis hin zu einer besseren Stressresistenz, Konzentration und Klarheit. Allein aufgrund dieser Vielzahl an Motiven haben Achtsamkeitstrainings im Laufe der letzten Jahre zusätzlich Aufmerksamkeit und Zuspruch erfahren [1]. Folglich werden sie mit einer Bandbreite an Slogans und Labels beworben [5], die damit teilweise sehr unterschiedliche Zielstellungen verbinden. Die Kommunikation von Achtsamkeitsförderung im Hochschulkontext hat somit zunächst mit der Herausforderung zu kämpfen, dass die Vorstellungen sehr unterschiedliche Facetten beinhalten.

Zudem speist sich die positive Wahrnehmung und die Motivation zur Teilnahme an Angeboten v. a. aus der eigenen Erfahrung mit Achtsamkeitstechniken [10]. Es gibt somit deutliche Unterschiede für den Zugang von Personen mit (umfangreichen) Vorerfahrungen (hier verstanden als *Achtsamkeitsprofis*) gegenüber Personen, die bislang noch keine Berührungspunkte mit Meditation und vergleichbaren Techniken haben (hier *Achtsamkeitslaien*). Aus anderen Gesundheitskampagnen ist bekannt, dass sich effektive Kommunikationsstrategien zur Steigerung von Interesse und Akzeptanz bei diesen verschiedenen Personengruppen wesentlich unterscheiden [3]. Es lässt sich daher vermuten, dass dies auch auf die Kommunikation von Achtsamkeitsangeboten zutrifft. Dementsprechend möchten wir mit unserer ersten Forschungsfrage (FF) ermitteln:

*FF1: Welche Vorstellungen verbinden Achtsamkeitsprofis und -laien mit Achtsamkeitsangeboten?*

Bei der Vielfalt der Vorstellungen zur Achtsamkeit birgt insbesondere der Aspekt der Spiritualität mögliche Konflikte für die Kommunikation im Hochschulsetting. Die Wurzeln der Achtsamkeitsförderung im spirituell-religiösen Bereich (wie etwa den Meditationspraktiken aus dem Buddhismus) können hierbei möglicherweise als Gegensatz zum rationalen und evidenzbasierten Anspruch der Universitäten an Bildung wahrgenommen werden. Assoziationen wie *spirituell, religiös* und *esoterisch* werden dabei häufig gleichgesetzt, ohne dass dies der Differenziertheit dieser Konzepte gerecht wird [6]. Der Medizinprofessor Jon Kabat-Zinn ist diesen Vorurteilen mit einem säkularisierten Curriculum zur „mindfulness-based stress reduction“ begegnet und hat damit das Thema *Achtsamkeit* im amerikanischen Gesundheitswesen verankert.

Inzwischen haben sich weitere Angebote im eher wissenschaftlich-empirischen Kontext etabliert. Dies sind insbesondere die achtsamkeitsbasierten Therapieprogramme (z. B. „mindfulness-based cognitive therapy“; [15]). Dennoch könnte u. a. der spirituelle Hintergrund den Zugang für Akademiker:innen und deren Motivation zur Teilnahme erschweren. Eine ausführliche Auseinandersetzung mit der Spiritualität und ihrem Spannungsfeld mit wissenschaftlichen Erkenntnisprozessen finden sich in [6, 9]. Über diese Herausforderung hinaus möchten wir weitere Chancen und Barrieren der Kommunikation von Achtsamkeitsangeboten aus Studierendenperspektive identifizieren, daher fragen wir:

*FF2: Welche Barrieren und Zugangsmöglichkeiten nehmen Studierende in der Kommunikation von Achtsamkeitsangeboten wahr?*

## Studiendesign und Untersuchungsmethoden

Um die Forschungsfragen zu beantworten wurden im Zeitraum vom 28.10.2020 bis 17.11.2020 dreizehn Studierende (Altersdurchschnitt 28 Jahre) leitfadengestützt interviewt. Das Sample wurde nach Geschlecht und Vorerfahrung zum Thema *Achtsamkeit* quotiert (s. Tab. 1), wobei *Achtsamkeitsprofis* Techniken wie Yoga, Pilates, Qui-Gong, autogenes Training, progressive Muskelentspannung oder Meditation regelmäßig in ihren Alltag integrieren und sich auch mit den theoretischen Hintergründen von *Achtsamkeit* beschäftigen, *Achtsamkeitslaien* dagegen über diese konkreten Erfahrungen nicht verfügen.

**Tab. 1:** Zusammensetzung des Samples

	Achtsamkeitsprofis (AP)	Achtsamkeitslaien (AL)
Männlich (m)	*n* = 3	*n* = 3
Weiblich (w)	*n* = 3	*n* = 4

Der Leitfaden enthielt Fragen zu den individuellen Vorstellungen, Motiven, Barrieren und Vorurteilen von *Achtsamkeit*. Aufgrund der pandemiebedingten Kontakteinschränkungen wurden die Interviews persönlich (*n* = 8), online (*n* = 3) und telefonisch (*n* = 3) durchgeführt. Für die Arbeit wurden Studierende der Universität Erfurt und Jena sowie der Fachhochschulen Erfurt und Gera aus sozial-, geistes-, sowie naturwissenschaftlich Studiengängen interviewt und die erhobenen Daten mithilfe des Programms MAXQDA (Version 2018) transkribiert und ausgewertet.

## Ergebnisse

### Vorstellungen zu Achtsamkeitsangeboten von Achtsamkeitsprofis und -laien

**Abb. 1:**
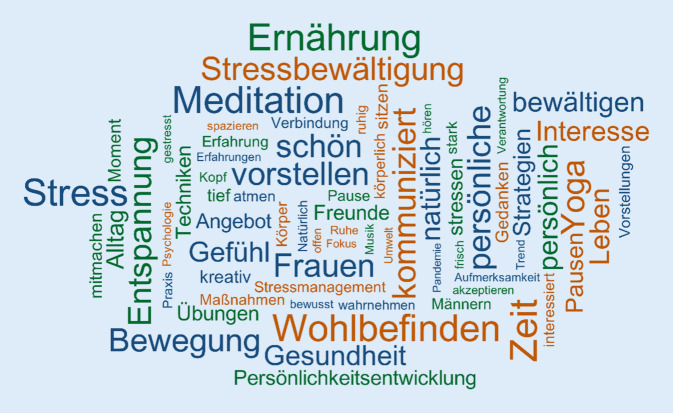
Wortwolke der Vorstellungen zu Achtsamkeitstrainings, mindestens 5‑mal genannt

Allen Gruppen gemeinsam war, dass *Achtsamkeit* als etwas Positives eingeschätzt wurde. Im Vordergrund stand dabei die Stressbewältigung, während die Persönlichkeitsentwicklung nur eine eher untergeordnete Rolle einnahm (s. Abb. 1). Das „Hauptverkaufsargument“ lautet: Achtsamkeit ist „die ultimative Waffe gegen Stress […] also etwas, das einfach jedem hilft“ (m, AL 3, Z: 37)^1^. Vor allem die *Achtsamkeitslaien* sahen darin eine Art der Aufmerksamkeit auf sich selbst, die anderen sowie die Umwelt. *Achtsamkeit* sehen sie als Teil einer gesunden und bewussten Lebensweise, verbunden mit Assoziationen wie „Nachhaltigkeit“, „Detox“, „Work-Life-Balance“, „Selbstbewusstsein“, „Selbstoptimierung“ und „Allheilmittel gegen Stress“. Ausdrücke wie „Resilienz“, „Selbstliebe“ und „Selbstwert“ wurden v. a. von *Achtsamkeitsprofis* genannt. Ihre Vorstellungen gingen über die „Selbstfürsorge“ hinaus und bezogen sich auch auf bestimmte Geisteszustände, erkenntnistheoretische und prosoziale Aspekte.

Gerade in der COVID-19-bedingten Pandemiesituation erschienen den Interviewten Achtsamkeitsangebote als eine hilfreiche Möglichkeit Stress zu bewältigen, zu lernen mit Unsicherheiten umzugehen und sich intensiv mit sich selbst auseinanderzusetzen: „da ich mich aus dem Stress ziehe und in die Ruhe gehe mit Achtsamkeit, kann es, glaube ich, auch helfen, um Ruhe zu bewältigen, sprich sich darin [der Coronasituation] nicht verloren und hibbelig zu fühlen.“ (m, AP 7, Z: 53). Als hilfreich wurden dabei insbesondere verschiedene digitale Angebote zum Thema *Achtsamkeit* empfunden, die genutzt werden, um sich mit anderen auszutauschen und sich zu motivieren, obwohl man dauerhaft zu Hause ist: „[…] der ganze Stress bleibt quasi an dem einen Ort, egal ob es der Arbeitsstress oder der Stress von zu Hause ist, das ist alles ein Ort […]. Und gerade da, wird dann denke ich mal, dann eher drauf geachtet, dass man mit Hilfe von Sport oder sonstigen Angeboten, die man zu Hause auch praktizieren kann,[…] dass dadurch halt auch dieses Gefühl, dass man zu Hause was machen kann, verstärkt und bestärkt wird […]“ (m, AL 2, Z: 106).

### Barrieren und Zugangsmöglichkeiten

*Achtsamkeit* wird oftmals mit Vorurteilen in Verbindung gebracht. *Achtsamkeit* wird als etwas wahrgenommen, dass „[…] mit einem selbst gar nichts zu tun hat oder mit der Welt wie sie funktioniert, und dass es nur für Leute interessant ist, die sich eh von vielen gesellschaftlichen Normen schon distanziert haben oder so.“ (w, AP 2, Z: 73). Es ist „[…] noch nichts Esoterisches, aber doch sowas ein bisschen Abgehobenes, ah ja, Meditation und spirituelle Spinner […]“ (m, AP 5, Z: 53).

Dieser Eindruck würde durch die Darstellung von *Achtsamkeit* in den Medien und der uneinheitlichen Anwendung des Begriffs zusätzlich noch verstärkt: „verallgemeinert halt viel, was teilweise aber sehr, sehr unterschiedlich auch ist, […] das führt zu Missverständnissen.“ (w, AP 1, Z: 89).

Insbesondere bei den männlichen *Achtsamkeitsprofis* scheint ein wissenschaftlich geprägter Zugang zum Thema wichtig zu sein: „[…] also ich habe halt auf anderem Weg und auch über andere Autoren zu dem Thema gefunden oder mich dafür begeistern können, so das Esoterische abschütteln können, aber so Begriffe wie Spiritualität haben da für mich doch noch einen Stellenwert, […]“ (m, AP 6, Z: 50) „[…] sobald irgendwas Esoterisches dazu kommt, dann ist es so eine Art nicht ernstzunehmende, religiöse Sache.“ (m, AP 6, Z: 54). Die *Achtsamkeitsprofis* wünschen sich daher eine Kommunikation der Hochschulangebote, die eher professionell und neutral ist und weniger esoterische Assoziationen weckt. Auf die Frage, wie Achtsamkeitsangebote vermittelt werden sollten, äußert eine Interviewpartnerin:

Also, dass es sehr professionell wirkt. Dass nicht so viele Zeichen oder Mandalas oder ähnliches drauf sind, die ihm so einen krassen Charakter geben, dass es wahrscheinlich relativ neutral formuliert und von der Farbgebung auch relativ neutral ist (w, AP 2, Z: 75.).

Man könnte auch genauso sagen, wir machen jetzt mal eine kurze Konzentrationspause [statt *Achtsamkeit*] und dann wäre es das gleiche (w, AP 1, Z: 85).

Insbesondere das im letzten Zitat angedeutete *Labeling *spiele eine entscheidende Rolle dabei. Studierende, so die Einschätzung der *Achtsamkeitsprofis*, würden dem Thema offener gegenüberstehen, wenn anstelle von achtsamkeitsbasierten Techniken eher von konzentrations- und performancesteigernden Übungen gesprochen würde. Diesen würde nicht so sehr dem Klischee der Esoterik oder Spiritualität nachhängen, dem viele Studierende oftmals kritisch gegenüberstehen: „Halt so einen aufrichtigen und auch einen nicht-esoterischen und ernsthaften Umgang mit dem Thema. Das ist das allerwichtigste, um das auf die Art und Weise schmackhaft zu machen.“ (m, AP 6, Z: 72).

Den *Achtsamkeitsprofis* war aber gleichzeitig noch wichtig, dass *Achtsamkeit* nicht kommerzialisiert wird: „also ich finde es schon problematisch, wenn aus Achtsamkeit so ein Geschäft gemacht wird, […] wenn es als Verkaufsargument genutzt wird, finde ich es schon blöd“ (w, AP 1, Z: 77). „Ich bin erstmal der Meinung, dass man es nicht so krass kommerzialisieren sollte. […] Also die Achtsamkeit nicht in den Dienst des Kapitalismus stellen“ (m, AP 6, Z: 56).

Sie befürchteten, dass *Achtsamkeit* zu stark instrumentalisiert wird und damit den Grundgedanken von *Achtsamkeit* im Kern verfehlt: „ich finde, oft wird Achtsamkeit unter so einem Selbstoptimierungsgedanken verkauft und das ist genau das, was ich gar nicht so sehe, sondern eher genau das Gegenteil, sondern eben einfach dieses Existieren an sich so“ (w, AP 1, Z: 59).

Als könnten wir unsere Leistung damit steigern. Da habe ich auch so ein bisschen meine Bedenken. Also sollen wir jetzt Achtsamkeitspraxen machen, um noch schneller Leistung bringen zu können in einer Gesellschaft, in einer Welt, die sowieso schon beschleunigt ohne Ende ist? (m, AP 6, Z: 60).

Als eine weitere wesentliche Barriere wurde das geringe Identifikationspotenzial genannt, welches die mediale Vermittlung von Achtsamkeitspraktiken insbesondere für Männer bietet:

Achtsamkeitskommunikation zeige überwiegend „Bilder(n) von irgendwelchen Frauen im mittleren Alter, die gerade zusammen Yoga machen und sich dabei alle bei Zoom oder Google Teams oder was weiß ich sehen und sich dann freuen, dass das ja alles so schön klappt und sie sich gegenseitig sehen können.“ (m, AL 3, Z: 39). Dagegen zeige sie nicht den *„[…] jungen männlichen Studenten, schwarz, weiß, wie auch immer, die da zusammen Yoga machen, sondern es ist halt die Frau im Alter meiner Mutter, die Yoga macht“* (m, AL 3, Z: 41).

Insbesondere eher dem weiblichen Geschlecht zugeschriebene Charaktereigenschaften wie Rücksicht, Emotionalität und Sensibilität tauchten in Verbindung mit dem Achtsamkeitsbegriff klassischerweise auf. Daher werde *Achtsamkeit* auch eher mit bestimmten* Studiengängen* in Verbindung gebracht. Neben den gesundheitswissenschaftlichen Studiengängen wurden auch Studierende aus den sozialwissenschaftlichen, pädagogischen und ökologischen Fachrichtungen als offener gegenüber Achtsamkeitsangeboten eingeschätzt. „Ich glaube schon, dass irgendwie sozialwissenschaftliche Studiengänge, [..], da offener für sind als jetzt die klassischen Ingenieure, Bautechniker oder ITler“ (w, AL 5, Z: 73). „Und in den Politikwissenschaften ist man mit Religion […], ja schon fast auf Kriegsfuß würde ich behaupten. Genau. So einfach dieser religiöse Touch, der das autonome und rationale Wesen versucht ruhig zu stellen“ (m, AP 6, Z: 54).

Für die *Achtsamkeitslaien* scheint zudem ein größerer Körperbezug bei der Vermittlung eine Möglichkeit für einen niedrigschwelligen Zugang zu bieten, da auch anderer Bewegungssport zur Stressbewältigung im Alltag angewendet wird. So wünscht sich etwa ein Interviewter:

Da jemanden zu finden, der wirklich in die Sparte passt, also sowohl kopflich mit einem reden kann und einem bei der Stressbewältigung helfen kann, und gleichzeitig den Sportaspekt mit reinbringt (m, AL 2, Z: 126).

Zur Frage nach der konkreten Integration der Angebote in den Studierendenalltag werden Bedenken gegenüber einer zu früh im Studium bzw. im Semester eingesetzten Kommunikation dieser Angebote geäußert. Der Start des Semesters und des Studiums sei häufig mit viel Aufregung, neuen sozialen Kontakten und Partys geprägt, die das Thema *Achtsamkeit* und Stressbewältigung eher in den Hintergrund treten lassen würden, während es im Verlauf des Semesters wieder wichtig wird, wenn die Prüfungen anstehen. Die Bedürfnisse der Studierenden würden am Anfang des Studiums eher im Bereich des Selbstmanagements liegen und im fortgeschrittenen Masterstudium eher in den Bereichen der Persönlichkeitsentwicklung.

Zudem bestand der Wunsch nach einer persönlichen und authentischen Vermittlung.

„Hauptsache die Person bringt ein, zwei interessante Gedankengänge mit, die mich sehr interessieren […] also irgendwas näher am Leben, etwas Praktischeres, was begeisterungsfähiger ist, als nur so eine Abarbeitung von Stichpunkten, was Persönliches“ (m, AP 7, Z: 67). „Und dann muss es auch vom Prof. glaubhaft sein und man muss es dem auch abnehmen; weil sonst funktioniert es halt erst gar nicht“ (w, AL 5, Z: 71).

## Diskussion

Die Ergebnisse verdeutlichen, dass Achtsamkeitsangebote an Hochschulen als evidenzbasiert, praxisnah und weltanschaulich kommuniziert werden sollten, damit Studierende sie als relevant wahrnehmen. Auch wenn zu den Ursprüngen des Achtsamkeitskonzepts eine Reihe spirituell geprägter Aspekte gehören (und diese Kontexte wesentlich sind), stehen insbesondere die Studierenden ohne Erfahrungen diesen Aspekten eher skeptisch gegenüber. Solche Erkenntnisse decken sich mit allgemeineren Diskussionen zur Etablierung von Achtsamkeitsangeboten an Hochschulen [6].

Zudem scheint es wichtig zu sein, die berufliche Relevanz des Achtsamkeitstrainings für die Studierenden im Zusammenhang mit der Persönlichkeitsentwicklung und somit der *Achtsamkeit* als Metabildung aufzuzeigen [2]. Die Forderung nach „professioneller Kommunikation“ bezieht sich somit hier nicht nur auf eine strategische und zielgruppenorientierte (und daher professionelle Kommunikation), sondern auch auf eine berufsorientierte Ausrichtung der Kommunikation.

Achtsamkeitsbasierte Angebote müssen nicht zwangsläufig mit dem Begriff „Achtsamkeit“ kommuniziert werden. Auch Begriffe wie *Kompetenzsteigerung, Selbstmanagement, Persönlichkeitsentwicklung *und *mentale und körperliche Entspannung *wären möglich [5]. Sie können zudem als etwas vermittelt werden, das sich nicht nur mental durchgeführt wird, sondern auch sportliche Aspekte berücksichtigt. Damit können insbesondere Studierende erreicht werden, die hinter dem Begriff *Achtsamkeit* eher eine esoterische Technik ohne Bezug zur Wirklichkeit vermuten. Von Achtsamkeitsprofis werden allerdings Begriffe wie Optimierung und Performancesteigerung kritisch gesehen. Ihnen ist wichtig, dass die Angebote nicht eine Leistungssteigerung (in einem kommerziellen Sinn) fokussieren, sondern eher ein Bewusstsein für den Umgang mit Herausforderungen im Alltag und im späteren Berufsleben anstreben. Hieraus wird deutlich, dass es bei der Kommunikation von Achtsamkeitsangeboten nicht um die Vermittlung aller potenziell möglichen Ziele, Inhalte und Vorstellungen von Achtsamkeitstrainings geht, sondern vielmehr die spezifische Betonung bestimmter Aspekte, die jeweils für die Zielgruppe relevant sind, den Zugang erleichtern kann.

Darüber hinaus vermitteln die Medien offenbar ein Bild der weiblich konnotierten Achtsamkeitsförderung. Eine Möglichkeit dies zu dekonstruieren, damit gerade auch männliche Studierende einen Zugang finden, bieten etwa Fallbeispiele von verschiedenen Studierenden mit unterschiedlichem Erfahrungslevel. Außerdem sollte die Kommunikation die Bedarfe der Zielgruppen zum passenden Zeitpunkt adressieren (wie vor und in der Prüfungszeit).

## Fazit für die Praxis

Zusammengefasst sollte die Kommunikation von Achtsamkeitsangeboten an Hochschulen weltanschaulich, vielseitig, authentisch, bedarfsorientiert im Zeitverlauf des Semesters und Studiums und berufsorientiert sein, damit möglichst viele Studierende sich angesprochen fühlen.

Dabei ist zu berücksichtigen, dass Kommunikation allein nicht ausreicht, sondern es auch der Förderung struktureller Rahmenbedingungen (Räume, Personal, Curricula etc.) bedarf. Daher sollten bspw. Mitarbeitende an Hochschulen als Nutzende sowie als Multiplikator:innen der Achtsamkeitsangebote in entsprechende Kommunikationskonzepte integriert werden.

## Footnotes

1 m = männlich/w = weiblich; AL = Achtsamkeitslaie/AP = Achtsamkeitsprofi; Z = Zeile.
